# Isolated Posttraumatic Lesion of the Ureter: A Rare Pathology

**DOI:** 10.7759/cureus.67437

**Published:** 2024-08-21

**Authors:** Marius Ivanuta, Dragos Puia, Catalin Pricop

**Affiliations:** 1 Urology, University of Medicine and Pharmacy "Grigore T. Popa", Iași, ROU; 2 Urology, "CI Parhon" Clinical Hospital, Iași, ROU

**Keywords:** endourology, jj stent, acute kidney failure, urinoma, ureteral trauma

## Abstract

Isolated traumatic ureteral injuries are uncommon physiological conditions that can be difficult to manage. This case concerns a 65-year-old man who was referred to the Urology Clinic, suffering left lumbar pain after falling from the same level. A computed tomographic scan revealed the presence of hydronephrosis and a significant perinephric extravasation. The patient underwent the insertion of a JJ stent due to acute renal failure, inflammatory syndrome, hydronephrosis, and the existence of a large urinoma. Postoperatively, remission of the inflammatory syndrome and acute renal failure was observed, and from a clinical point of view, the patient became asymptomatic. Three months after the surgery, the imaging studies show no urine leakage outside the urinary system.

## Introduction

Spontaneous ureteric rupture is a highly uncommon medical condition where urine leaks out of the ureter without any apparent traumatic cause. This phenomenon is characterized by the extravasation of urine, which can lead to significant complications if not promptly addressed. Several underlying conditions have been associated with this rare event. Notably, documented cases have identified factors such as pregnancy, which can cause increased pressure on the urinary tract; ureteral stenosis, which narrows the ureter and obstructs urine flow; tumors, which may invade or compress the ureter; bladder outlet obstruction, which can lead to backpressure and ureteral damage; and retroperitoneal fibrosis, a condition that leads to the formation of excessive fibrous tissue around the ureter, causing constriction. Each of these conditions can contribute to the spontaneous rupture of the ureter, highlighting the complexity and multifactorial nature of this medical issue [1,2]. Ureteral injury resulting from external trauma is uncommon, with a prevalence of less than 4% in cases of penetrating trauma and less than 1% in situations of blunt trauma. Posttraumatic ureteral rips are uncommon because the ureter is situated deeply in the retroperitoneum and protected by substantial connective tissue and laterovertebral muscles. Leakage of urine resulting from ureteral injury can lead to the formation of a urinoma, which is defined as a localized accumulation of urine within the peritoneal or retroperitoneal space. This pathological urine collection creates an environment conducive to microbial proliferation, which can result in subsequent infections [3]. Beyond the risk of infection, urine outside anatomical pathways can irritate the surrounding structures, including the intestines and peritoneum. This irritation can manifest as abdominal pain and may contribute to the development of paralytic ileus. Additionally, the extravasated urine can induce inflammation of the peritoneal cavity, potentially leading to more severe complications, such as peritonitis, which is an acute inflammatory response of the peritoneum. These effects can significantly complicate the clinical scenario, necessitating comprehensive medical management and intervention to address the primary injury and its associated complications [4].

## Case presentation

**Figure 1:**
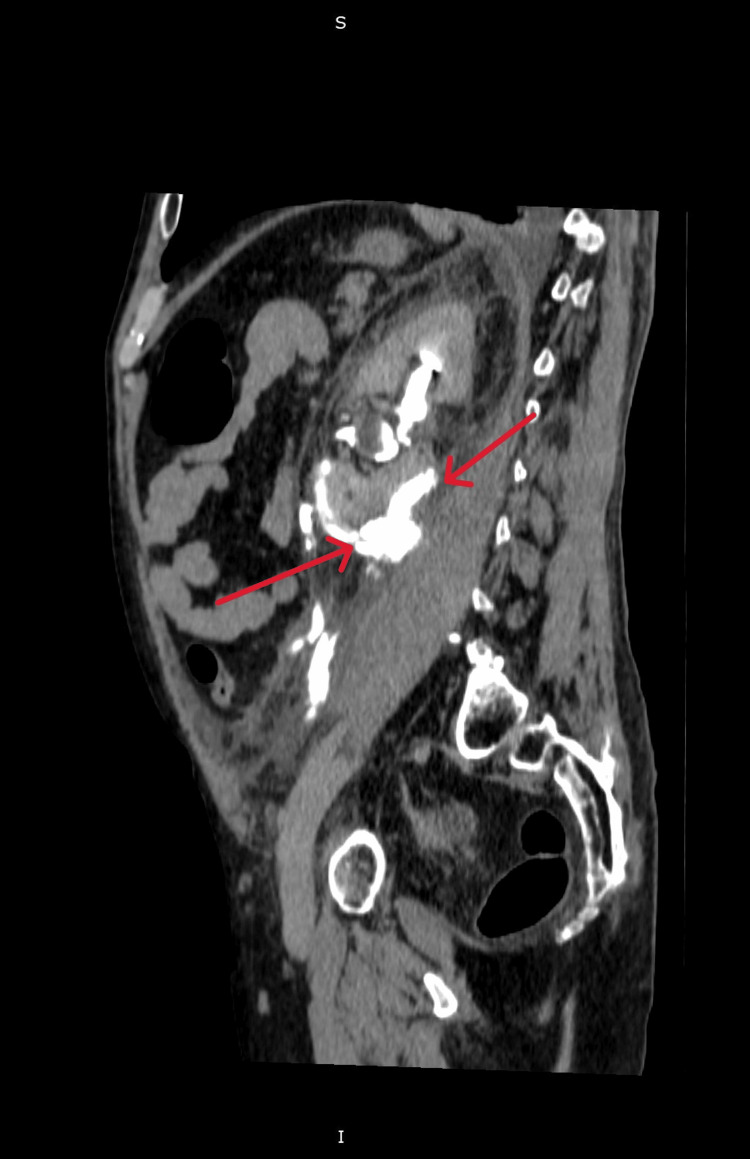
Contrast-enhanced CT image showing left hydronephrosis and large urinoma The arrows indicate the presence of urinary extravasation as well as the large perinephric collection.

A 65-year-old man without any notable urological background has been examined in the emergency unit for pain in the left flank and dizziness. These symptoms occurred after he fell from the same level following alcohol consumption. Given the patient's high blood pressure values (210/110 mmHg), admission to the Internal Medicine Clinic was decided, and antihypertensive therapy started. On the second day post-admission, the patient develops exacerbated pain symptoms, motivating the decision to perform a contrast-enhanced abdominal-pelvic tomography. The tomographic evaluation revealed the presence of a large perirenal extravasation (Figure 1), which is why the patient was admitted to the Urology Clinic.

Diagnostic assessment

Upon admission to the Urology Clinic, the patient is hemodynamically and respiratorily stable, presenting significant left lumbar pain. Table 1 shows that laboratory analyses revealed an important inflammatory syndrome and acute renal failure.

**Table 1:** Laboratory findings at admission and discharge Cl: serum chloride, Cr: serum creatinine, CRP: C-reactive protein, Hb: hemoglobin, K: serum potassium, Na: serum sodium, PLT: thrombocyte, Ur: serum urea, WBC: white blood cell

Blood test	At admission	Upon discharge	Normal values
WBC	14.79 * 10^3^/µL	8.23 * 10^3^/µL	4-10 * 10^3^/µL
Hb	13g/dL	13g/dL	13-17.3 g/dL
PLT	203 * 10^3^/µL	28,610^3^/µL	150-450 * 10^3^/µL
Cr	1.92 mg/dL	0.83 mg/dL	0.7-1.3 mg/dL
Ur	56 mg/dL	21 mg/dL	10-50 mg/dL
CRP	201.2 mg/L	19 mg/L	0-5 mg/L
K	3.8 mmol/L	4.41 mmol/L	3.5-5.1 mmol/L
Na	126 mmol/L	139 mmol/L	135-148 mmol/L
Cl	91 mmol/L	103.2 mmol/L	98-107 mmol/L

**Figure 2:**
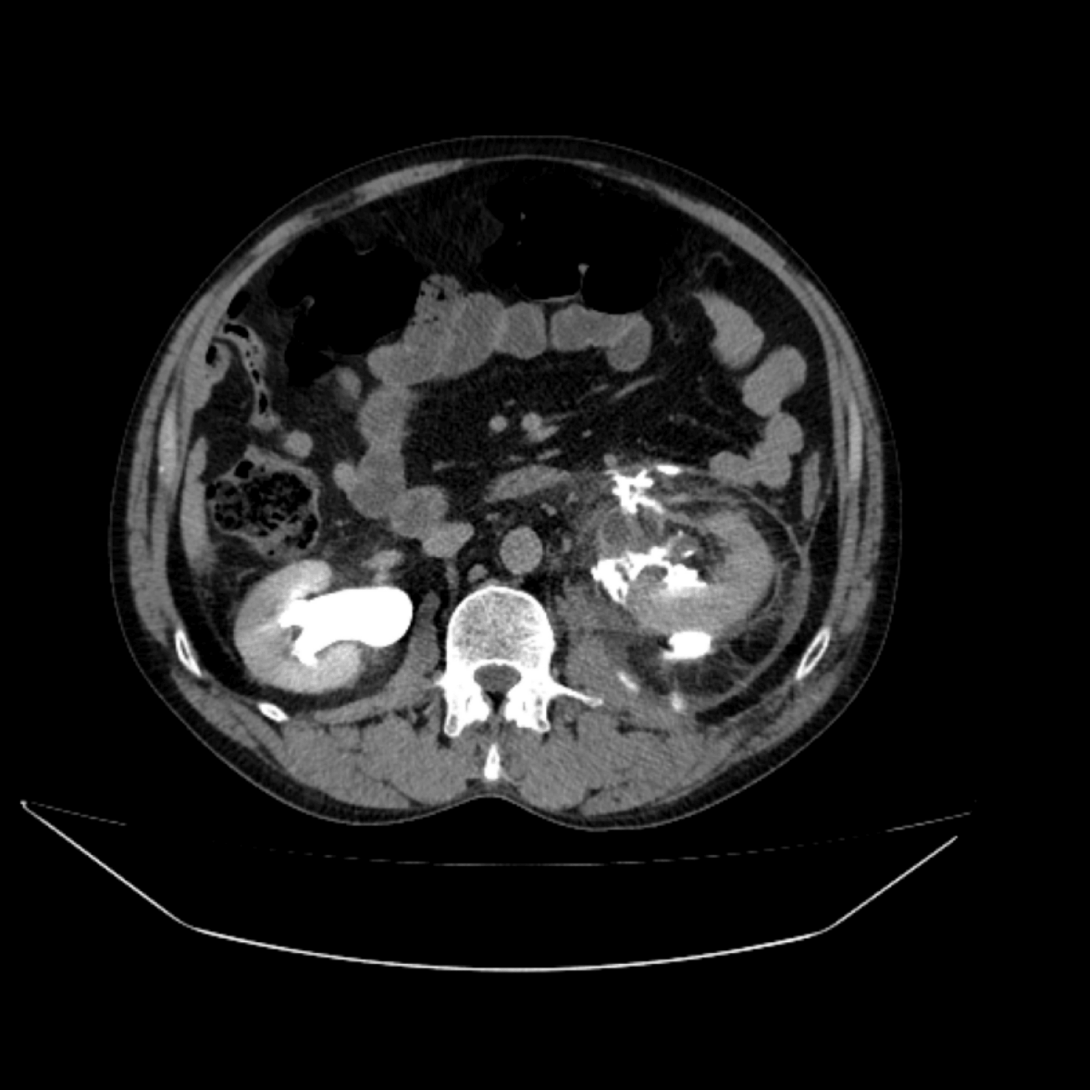
Contrast-enhanced CT image showing the presence of urinary extravasation at the level of the left upper lumbar ureter

The contrast-enhanced CT revealed the existence of contrast leakage at the upper lumbar ureter, along with a sizeable fluid-filled mass in the perinephric area measuring 11 cm in diameter, extending into the left iliac fossa and important hydronephrosis (Figure 2).

Therapeutic interventions

**Figure 3:**
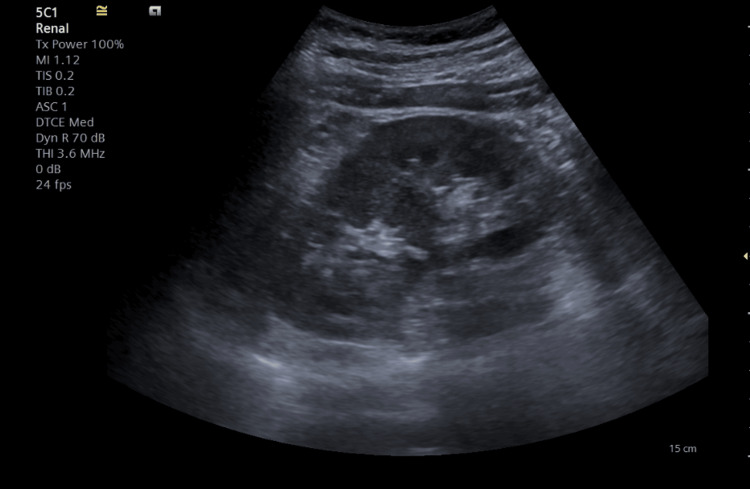
Ultrasound image demonstrating the absence of urinary extravasation

Preoperatively, antibiotic treatment with Ceftriaxone 1 g two hours before surgery was initiated. Retrograde ureteropyelography proved the extravasation of the contrast substance at the level of the left pyeloureteral junction. A hydrophilic guidewire was advanced in the left superior calyceal group, and a JJ ureteral catheter was subsequently attempted, which failed. In this context, a simple ureteral stent was placed. The biological balance on the second postoperative day demonstrated the improvement of renal function and the inflammatory syndrome, and from a clinical point of view, the patient became asymptomatic. The ultrasound on the third postoperative day highlighted the complete remission of perinephric fluid collection and hydronephrosis (Figure 3).

**Figure 4:**
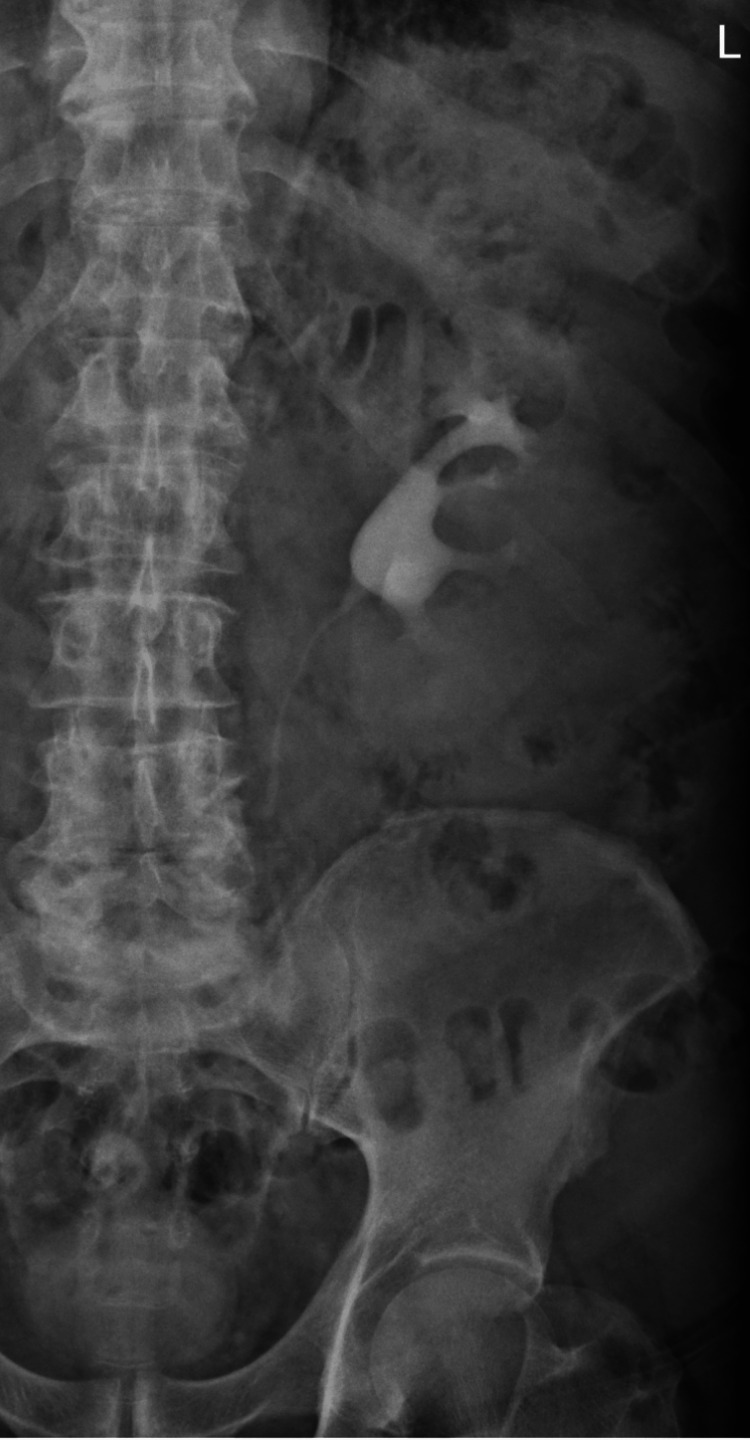
Urographic image after the removal of the JJ stent demonstrating the absence of extravasation of the contrast substance

Surgical reintervention was decided on the third postoperative day, and a JJ stent was placed successfully. The postoperative evolution was good, and the patient was discharged with normal renal function and no inflammatory syndrome. Twelve weeks after the first admission, the patient returned to the Urology Clinic, where the JJ stent was removed, and a control urography was performed, which revealed the absence of urinary extravasation (Figure 4).

## Discussion

Ureteral rupture may occur either spontaneously or as a result of traumatic injury. Traumatic ureteral injuries are relatively rare due to the significant anatomical protection afforded by the retroperitoneal space [5]. The ureter is encased by several protective structures, including the bony pelvis, the psoas muscles, surrounding adipose tissue, and the vertebral column.

On the left side, the ureteropelvic junction is positioned posterior to the pancreas and the ligament of Treitz, which further shields it from direct trauma. Additionally, the inferior mesenteric artery and the sigmoidal arteries intersect anteriorly at the lower portion of the left ureter, providing an additional layer of anatomical protection. Conversely, on the right side, the ureter is posterior to the duodenum and laterally adjacent to the inferior vena cava. In this region, the right colic and ileocolic veins pass anteriorly to the ureter, adding to the protective environment. This complex arrangement of anatomical structures helps minimize the incidence of traumatic injuries to the ureter, although such injuries, when they do occur, can lead to significant clinical complications [6].

Due to this organ's retroperitoneal location, isolated ureteral injuries represent rare pathological entities. Most often, the ureter is iatrogenically injured during pelvic surgery or urological endoscopy. However, other non-iatrogenic causes of ureteral lesions are described in the literature [7]. Computer tomography imaging is crucial for diagnosing and determining the severity of ureteral injuries. In addition, retrograde ureteropyelography may successfully detect the extravasation of the contrast substance and determine the specific location of the ureteral injury [8].

The treatment of ureteric rupture lacks standardization and varies from conservative approaches, such as nephrostomy and ureteric stenting, to more invasive interventions, including ureteric reconstruction and nephroureterectomy in severe cases [9,10]. We decided to use endourological techniques to manage the injury to the ureter in our patient. Despite initial difficulties in inserting the auto-static stent, the insertion of a JJ stent was successful, using a simple ureteral catheter to decrease local inflammation and pressure.

## Conclusions

Isolated post-traumatic ureteral injuries are infrequent and often present with non-specific symptoms, making accurate diagnosis challenging. This case report illustrates the successful management of such an injury through endoscopic techniques, specifically the retrograde placement of a JJ stent. The patient’s favorable outcome, characterized by the resolution of inflammatory symptoms, improvement in renal function, and absence of urinary extravasation upon follow-up, highlights the effectiveness of endoscopic treatment. This approach not only facilitated a significant reduction in the need for more invasive surgical interventions but also minimized associated risks and recovery time. The findings underscore the importance of considering endoscopic management for isolated ureteral injuries as a viable option, providing excellent outcomes and optimizing patient care.

## References

[1] Low LS, Nair SM (2020). Spontaneous distal ureteric rupture: a rare case report and review of literature. Asian J Urol.

[2] Pace K, Spiteri K, German K (2017). Spontaneous proximal ureteric rupture secondary to ureterolithiasis. J Surg Case Rep.

[3] Lai CJ, Chang MY, Huang PC, Chu YC (2019). Complete ureter avulsion causing a long defect as a complication of posterior spine fusion: a rare case treated with nonrobotic laparoscopic repair. Res Rep Urol.

[4] Pampana E, Altobelli S, Morini M, Ricci A, D'Onofrio S, Simonetti G (2013). Spontaneous ureteral rupture diagnosis and treatment. Case Rep Radiol.

[5] Ay D, Yencilek E, Celikmen MF, Akkas M, Ekci B (2012). Spontaneous rupture of ureter: an unusual cause of acute abdominal pain. Am J Emerg Med.

[6] Jackson LA, Ramirez DM, Carrick KS, Pedersen R, Spirtos A, Corton MM (2019). Gross and histologic anatomy of the pelvic ureter: clinical applications to pelvic surgery. Obstet Gynecol.

[7] Pereira BM, Ogilvie MP, Gomez-Rodriguez JC (2010). A review of ureteral injuries after external trauma. Scand J Trauma Resusc Emerg Med.

[8] Siram SM, Gerald SZ, Greene WR (2010). Ureteral trauma: patterns and mechanisms of injury of an uncommon condition. Am J Surg.

[9] Alabousi A, Patlas MN, Menias CO (2017). Multi-modality imaging of the leaking ureter: why does detection of traumatic and iatrogenic ureteral injuries remain a challenge?. Emerg Radiol.

[10] Chen GH, Hsiao PJ, Chang YH (2014). Spontaneous ureteral rupture and review of the literature. Am J Emerg Med.

